# Low levels of HIV-1 drug resistance mutations in patients who achieved viral re-suppression without regimen switch: a retrospective study

**DOI:** 10.1186/s12866-020-1706-1

**Published:** 2020-01-20

**Authors:** Chika K. Onwuamah, Jonathan Okpokwu, Rosemary Audu, Godwin Imade, Seema T. Meloni, Azuka Okwuraiwe, Philippe Chebu, Adesola Z. Musa, Beth Chaplin, Ibrahim Dalhatu, Oche Agbaji, Jay Samuels, Oliver Ezechi, Mukhtar Ahmed, Georgina Odaibo, David O. Olaleye, Prosper Okonkwo, Babatunde Lawal Salako, Elliot Raizes, Chunfu Yang, Phyllis J. Kanki, Emmanuel O. Idigbe

**Affiliations:** 1 Microbiology Department, Centre for Human Virology and Genomics, Nigerian Institute of Medical Research, 6 Edmund Crescent, Yaba, Lagos, 101212 Nigeria; 2 Jos University Teaching Hospital, Jos, Plateau State Nigeria; 3 Harvard T. H. Chan School of Public Health, Boston, MA USA; 4 APIN Public Health Initiative Nigeria, Ltd./Gte, Abuja, FCT Nigeria; 5 Centers for Disease Control-Nigeria, Abuja, FCT Nigeria; 6 University of Ibadan, Ibadan, Oyo State Nigeria; 7 Centers for Disease Control and Prevention, Atlanta, GA USA

**Keywords:** Drug resistance mutation, Adherence, Re-suppression, Virologic failure

## Abstract

**Background:**

We identified a HIV-positive cohort in virologic failure (VF) who re-suppressed without drug switch. We characterized their drug resistance mutations (DRM) and adherence profiles to learn how to better manage HIV drug resistance.

A retrospective cohort study utilizing clinical data and stored samples. Patients received ART at three Nigerian treatment centres. Plasma samples stored when they were in VF were genotyped.

**Result:**

Of 126 patients with samples available, 57 were successfully genotyped. From ART initiation, the proportion of patients with adherence ≥90% increased steadily from 54% at first high viral load (VL) to 67% at confirmed VF, and 81% at time of re-suppressed VL. Sixteen (28%) patients had at least one DRM. Forty-six (81%) patients had full susceptibility to the three drugs in their first-line (1 L) regimen. Thirteen (23%) were resistant to at least one antiretroviral drug but three were resistant to drugs not used in Nigeria. Ten patients had resistance to their 1 L drug(s) and six were fully susceptible to the three drugs in the recommended second-line regimen.

**Conclusion:**

This cohort had little drug resistance mutations. We conclude that if adherence is not assured, patients could exhibit virologic failure without having developed mutations associated with drug resistance.

## Background

For treatment of HIV-1 in resource-limited settings, the World Health Organization (WHO) specifies that if viral load (VL) is > 1000 copies per millilitre (cp/mL) after 6 months on treatment, virologic failure (VF) is suspected [1]. In this scenario, adherence support is recommended by the Nigerian national guideline along with treatment of any opportunistic infections followed by reassessment in 3 months for clinical and laboratory parameters. If improvement is noted, the patient is continued on their first-line (1 L) regimen. However, if there is no improvement, a second VL test is performed. Per guidelines, patients with a second VL ≥1000 cp/mL are switched to a second-line (2 L) regimen [1].

The emergence of HIV drug resistance mutations (DRMs) is influenced by many factors, foremost of which is adherence to antiretroviral therapy (ART) [2, 3]. Adherence to ART can be measured by various methods [4], including MEM system [5], face-to-face interviews [2, 3], self-reported adherence [6], review of pharmacy refill pick-ups, [7] and measuring blood or hair antiretroviral (ARV) levels [8]. It has long been accepted in clinical practice that an intermediate level of adherence at 70–89% is associated with higher risks of VF and detection of DRMs as compared with patients having high (≥90%) or low (< 70%) levels of cumulative adherence [2]. In addition, increased failure rates on second-line regimens have been reported in sub-Saharan Africa, mostly due to non-adherence to treatment [9].

We discovered a cohort of patients who met the criteria for virologic failure but surprisingly re-suppressed VL without a change of regimen. At present, there is little data regarding DRMs in patients that re-suppress VL following confirmed VF, particularly in the context of adherence patterns. Thus, we conducted this evaluation to examine the range of HIV DRMs, drug resistance and adherence patterns in this cohort. We believe findings will guide management of HIV drug resistance, especially in resource-constrained settings that have limited drug options.

## Results

### Demographics

**Fig. 1:**
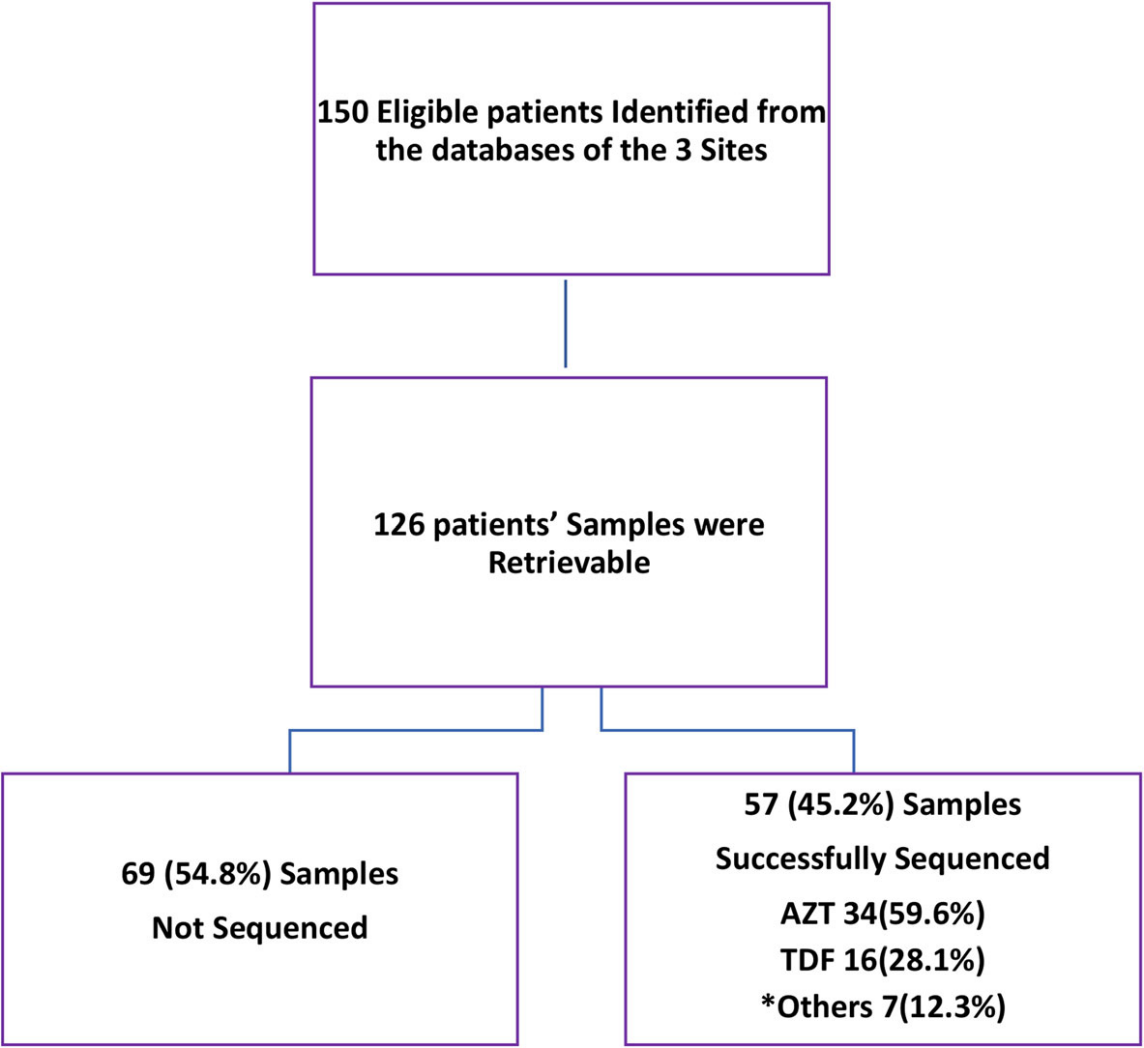
Study Consort Diagram. TDF = Tenofovir; AZT = Zidovudine; *Others = Backbone NRTI was switched

In total, 126 patients who met the inclusion criteria for the study and had remnant samples available were tested (Fig. 1). Among the 126 samples tested, 57 (45%) were successfully genotyped and included in the final analysis. There was no significant difference in demographics and viral load between those successfully sequenced and those not sequenced (Table 1). Of the 57 patients, 39 (68%) were female and the median age was 34 years (interquartile range (IQR): 30.0–41.5; Table 2). Thirty-four patients (60%) were on zidovudine (AZT)-based 1 L regimens while 16 (28%) were on tenofovir (TDF)-based 1 L regimens (Table 2). Seven (12%) patients had substitutions in their original 1 L backbone regimen and were classified as “other” 1 L regimens. The major subtypes represented in the cohort were subtype G (44%) and CRF02_AG (40%). Except for sex (*p* = 0.042), none of the other variables were significantly associated with drug resistance in the bivariate analyses (Table 2).

**Table 1:** Comparison of Viral load (VL) between samples not sequenced (*n* = 69) and those successfully genotyped (*n* = 57)

	Option	Sample Bleed Year					Total
		2006	2007	2008	2009	2010	
Genotyping Successful?	No	6	5	12	27	19	69
	Median VL	3250.5	14,467.0	4861.0	6499.3	6222.9	
	Yes	4	3	3	23	24	57
	Median VL	4356.5	13,703.0	33,500.0	15,044.2	5418.9	
Sample Retrieval	No	0	2	14	7	1	24
Total							150

**Table 2:** Characteristics* of the patients successfully genotyped (*N* = 57)

Category	Parameters	Characteristics	Total [N (% or IQR)]	^#^*P*-Values
Demographics	Sex	Female	39 (68)	0.042
		Male	18 (32)	
	Age at baseline	Median (IQR) years	34 (30–42)	0.921
	Marital Status	Single	12 (21)	0.663
		Married	29 (51)	
		Divorced/Separated	4 (7)	
		Widowed	12 (21)	
	Education	None	6 (11)	0.770
		Primary	14 (25)	
		Secondary	18 (32)	
		Tertiary	19 (33)	
	Occupation	Not Employed	15 (26)	0.338
		Employed	42 (74)	
Clinical Parameters	First-line Backbone NRTI	AZT	34 (60)	0.978
		TDF	16 (28)	
		^b^Others	7 (12)	
	First-line Second NRTI	3TC	39 (68)	0.666
		FTC	18 (32)	
	First-line NNRTI	NVP	43 (75)	0.071
		EFV	14 (25)	
	Time on First-line ART (Months)	ART Initiation to F_1_	21 (12–36)	0.217
		F_1_ to F_C_	5 (4–6)	0.086
		F_C_ to Viral Re-suppression	5 (3–10)	0.118
Laboratory Parameters	Baseline CD4+ cells/μL	< 200	38 (67)	0.780
		200–350	17 (30)	
		> 350	2 (4)	
		Median (IQR)	155 (105–235)	
	Baseline Viral Load (VL), copies/mL	≤100,000	36 (63)	0.774
		> 100,000	18 (32)	
		Unknown	3 (5)	
		Median (IQR)	43,587 (13128–176,990)	
	VL at initial failure (copies/mL)	Median (IQR)	9113 (3680–49,670)	0.768
	VL at confirmatory failure (copies/mL)	Median (IQR)	16,266 (2042–4,002,513)	0.454
	HIV-1 Subtype	G	25 (44)	0.261 *Grouped as G, CRF02_AG and Others*
		CRF02_AG	23 (40)	
		A	3 (5)	
		CRF06_cpx	3 (5)	
		J	1 (2)	
		C	1 (2)	
		Recombinant of A1, G	1 (2)	

^a^Demographic characteristics of those successfully genotyped were not significantly different from those not genotyped; *F*_*1*_ First VL ≥ 1000 cp/mL; *F*_*C*_ Second VL ≥ 1000 cp/mL. ^**#**^*P*-value is for difference in patient characteristic and drug resistance. *IQR* Interquartile range. ^b^Others = Backbone NRTI was switched

### Drug resistance mutations

Among the 57 patients with genotype data available, 16 (28%) had at least one HIV-1 DRM (Table 4). Four patients (7%) had DRMs to NRTIs while 14 (25%) had DRMs to non-nucleoside reverse transcriptase inhibitor (NNRTIs). Of the 16 patients with at least one HIV-1 DRM, four (7% of 57) patients had DRMs to both NRTIs and NNRTIs. Two patients (4% of 57) had DRMs to protease inhibitors (PIs): one patient had M46 L (major PI DRM) while the other patient had L23I (minor PI DRM). In addition, 51 (90%) patients had the K20I polymorphism, which is a consensus amino acid in subtypes G and CRF02_AG. Only one patient had a thymidine analogue mutation.

### Adherence, HIV drug resistance mutations and viral load

**Fig. 2:**
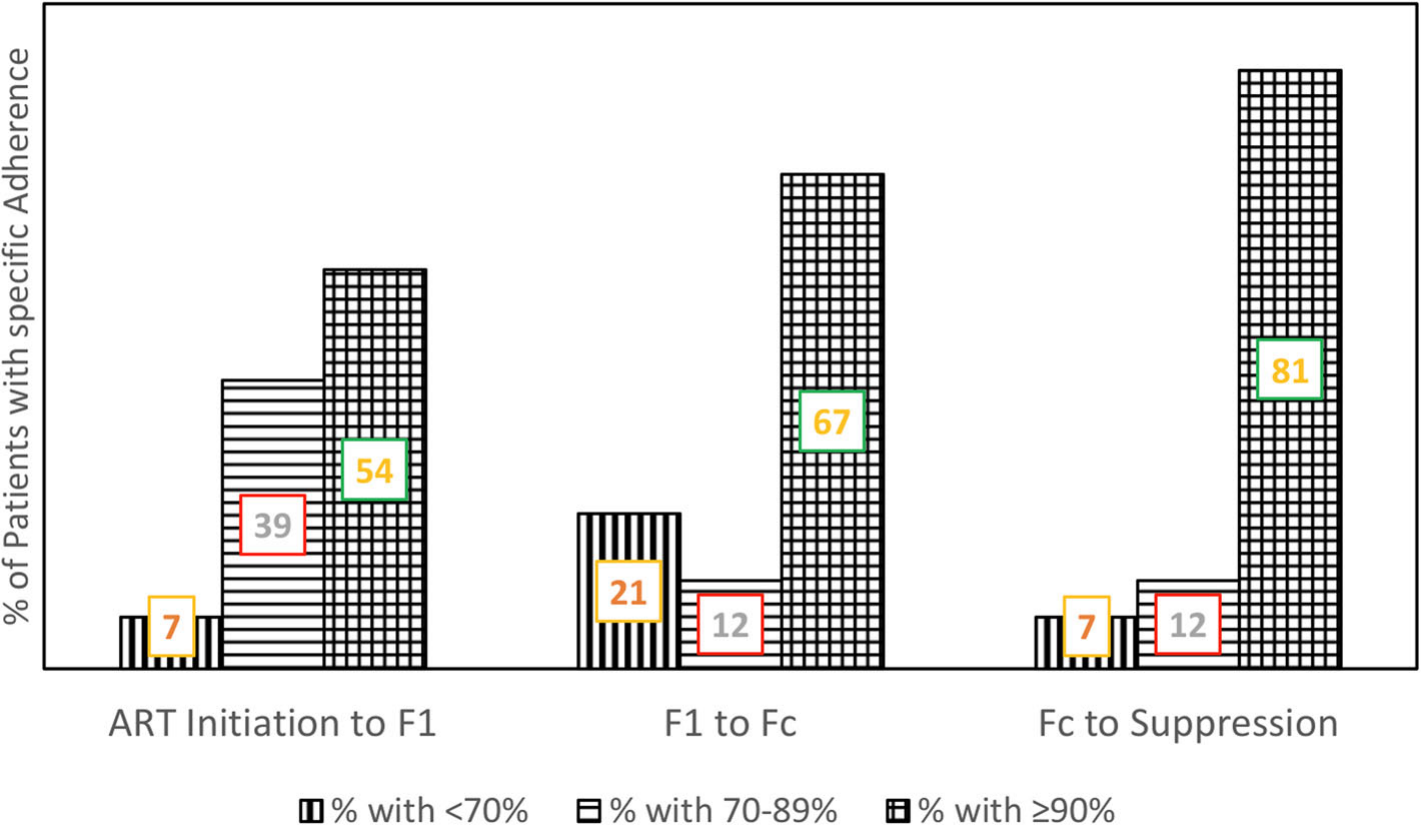
Pattern of improvement in adherence from ART initiation till viral re-suppression. AI to F1: Period between ART Initiation to the first VL above 1000 cp/mL (F_1_);**.**F1 to F_C_: Period between the first VL above 1000 cp/mL (F_1_) and confirmatory VL above 1000 cp/mL (F_C_);**.**F_C_ to Suppression: Period between the confirmatory VL above 1000 cp/mL (F_C_) and viral re-suppression

In the evaluation of adherence patterns from ART initiation to the first VL ≥ 1000 cp/mL (F_1_); F_1_ to the second VL ≥ 1000 cp/mL (F_C_); F_C_ to VL re-suppression, the proportion of patients with adherence ≥90% increased steadily (Fig. 2). Stratified into < 70%, 70–89% and ≥ 90%, adherence between ART initiation to F_1_ only was significantly associated with drug resistance (*p* = 0.037) as six of eight patients with drug resistance to at least one drug had 70–89% adherence. This association was specifically with only NRTI resistance and the M184 V mutation (Table 3). However. individual changes in adherence were not significantly associated with the detection of DRMs nor drug resistance. Median VL was also significantly higher for those with median adherence < 90% but at F_1_ only (Table 4).

**Table 3:** Drug Resistance Patterns by Median Adherence Prior to F_1_

Adherence from ART Initiation to F_1_	Total [n (%)]	≥1 DRM	Resistance to NRTI	Resistance to NNRTI	Resistance to M184 V
< 70%	4 (7)	7%	0%	8%	0%
70–89%	22 (39)	50%	100%	58%	100%
≥ 90%	31 (54)	43%	0%	33%	0%
	p-value	0.583	0.033^a^	0.245	0.033^a^

*ART* Antiretroviral therapy, *F*_*1*_ First VL ≥ 1000 cp/mL. ^a^Statistically significant

**Table 4:** Median Viral Load (VL) by Median Adherence

Viral Load	Adherence			*P*-Value
	< 70%	< 70–89%	≥ 90%	
Median VL at F_1_	94,670 IQR: 15902–271,109	19,833 IQR: 5822–103,041	4745 IQR: 3565–17,610	0.021^a^
Median VL at F_C_	30,439 IQR: 5466–96,682	16,266 IQR: 4471–51,973	20,201 IQR: 4107–74,390	0.950

*F*_*1*_ First VL ≥ 1000 cp/mL, *F*_*C*_ Second VL ≥ 1000 cp/mL, *IQR* Interquartile range

### Predicted drug susceptibility profiles among this cohort

Despite confirmed VF and the presence of DRMs in some patients, most of the patients (*n* = 46; 81%) were fully susceptible to their 1 L regimens, including six patients with detectable DRMs. Of all the 16 patients with DRMs, only thirteen (23% of 57) patients had resistance to any ARVs (Table 5; patients 3, 5–16). Of these thirteen, four patients (7% of 57) retained susceptibility to only one drug in their current 1 L regimen, ranging from intermediate-level resistance (*n* = 1), low-level resistance (*n* = 1) to susceptible (*n* = 2; Table 5; patients 13–16). All patients were susceptible to lopinavir/ritonavir (LPV/r) and atazanavir/ritonavir (ATV/r), the two PIs recommended for 2 L regimens in Nigeria, while only one patient had less than two 2 L drugs to which they were susceptible (GSS = 1.5). Of the 13 patients with resistance to at least one drug, eight had GSS = 3 for the recommended 2 L regimen. Three of the four patients with DRMs to NRTI were on an AZT-based 1 L regimen and the fourth patient was on a TDF-based 1 L regimen (Table 5). Nine out of the 14 patients with DRMs to NNRTI were on NVP-based ART, while the remaining five patients were receiving EFV-based regimens (Table 5). Drug susceptibility patterns for the entire cohort can be seen in Additional file 1.

**Table 5:** Drug Susceptibility Profiles of patients with HIV Drug Resistance Mutations Detected

Patient No.	Regimen	Drug Resistance Mutation Types			Susceptibility to 1 L Drugs									Susceptibility to 2 L Drugs			2 L GSS	Subtype
		NRTIs	NNRTIs	PIs	ABC	AZT	FTC	3TC	TDF	EFV	ETR	NVP	RPV	DRV/r	ATV/r	LPV/r		
1	^b^Others/FTC/NVP	None	V90I	K20I	S	S	^a^S	S	^a^S	S	S	^a^S	S	S	S	S	3.0	02_AG
2	TDF/FTC/NVP	None	V90IV	K20I	S	S	^a^S	S	^a^S	S	S	^a^S	S	S	S	S	3.0	02_AG
3	AZT/3TC/EFV	None	None	M46 L, K20I	S	^a^S	S	^a^S	S	^a^S	S	S	S	S	S	S	2.75	G
4	^#^Others/3TC/EFV	None	None	L10I, L23IL	S	^a^S	S	^a^S	S	*S	S	S	S	S	S	S	3.0	A1
5	AZT/3TC/NVP	None	E138EG	None	S	^a^S	S	^a^S	S	S	S	*S	L	S	S	S	3.0	C
6	TDF/FTC/EFV	None	K103Q, E138AE	K20I	S	S	^a^S	S	^a^S	^a^S	S	S	L	S	S	S	3.0	G
7	AZT/3TC/NVP	None	V90IV, V108IV	None	S	^a^S	S	^a^S	S	S	S	^a^L	S	S	S	S	3.0	J
8	AZT/3TC/NVP	None	V108I	K20I	S	^a^S	S	^a^S	S	S	S	^a^L	S	S	S	S	3.0	G
9	AZT/3TC/EFV	None	K103 N	K20I	S	^a^S	S	^a^S	S	^a^H	S	H	S	S	S	S	3.0	G
10	AZT/3TC/EFV	None	V106A, F227 L	K20I	S	^a^S	S	^a^S	S	^a^H	S	H	S	S	S	S	3.0	02_AG
11	^b^Others/FTC/NVP	None	K103KN	K20I	S	S	^a^S	S	^a^S	H	S	^a^H	S	S	S	S	3.0	02_AG
12	TDF/FTC/NVP	None	V90I, K103 N	K20I	S	S	^a^S	S	^a^S	H	S	^a^H	S	S	S	S	3.0	02_AG
13	AZT/3TC/NVP	M184 V	K103 N, E138A	K20I	L	^a^S	H	^a^H	S	H	S	^a^H	L	S	S	S	2.0	G
14	AZT/3TC/EFV	D67DN, K70R, M184 V, K219E	K103 N, V108I	K20I	L	^a^L	H	^a^H	L	^a^H	S	H	S	S	S	S	1.5	02_AG
15	TDF/3TC/EFV	K70E, M184 V	K103 N, V108I, H221Y	L10I, K20I	L	S	H	^a^H	^a^L	^a^H	S	H	S	S	S	S	2.0	02_AG
16	AZT/3TC/NVP	M184 V	A98AG, K101E, Y181C	K20I	L	^a^S	H	^a^H	S	L	L	^a^H	H	S	S	S	2.0	02_AG

*S* Susceptible and Potential low-level resistance, *L* Low-level resistance and Intermediate-level resistance, *H* High-level resistance. *NRTIs* Nucleoside Reverse Transcriptase Inhibitors, *NNRTIs* Non-Nucleoside Reverse Transcriptase Inhibitors, *PIs* Protease Inhibitors; 1 L-First-line; 2 L = Second-line: *GSS* Genotype Sensitivity Score. ^a^Indicate drugs in patient’s First-line regimen; ^b^Others = Backbone NRTI was switched

## Discussion

In this study, we determined DRM, drug resistance and adherence profiles of ART patients with confirmed VF who re-suppressed their VL in the absence of a regimen switch. The difference in sex is related to the higher number of females in the treatment cohorts at these centres as they have better treatment-seeking behaviour than men. Sixteen (28%) patients in this cohort had at least one HIV DRM, but only 13 (23%) were resistant to at least one drug. Of the 13 patients with resistance to at least one drug, all were susceptible to the PIs recommended for 2 L regimens in Nigeria.

We find that most patients failed and re-suppressed without developing DRMs. In addition, those who had DRMs were still able to re-suppress VL. Firstly, it is not surprising that patients without DRMs re-suppressed VL. Secondly, we note that some patients with functional monotherapy were still able to re-suppress VL, reiterating that the presence of DRMs itself does not necessarily predicts VF. However, its noteworthy that all four patients with dual-class DRMs (NRTI and NNRTIs), with only their NRTI backbone being sensitive, had the M184 V mutation. The M184 V mutation reduces viral replication, increases susceptibility to AZT and TDF, and thus slow emergence of VF to these drugs. These effects could be partly responsible for viral re-suppression especially in these four with several DRMs.

Improved adherence appears to have helped them achieve re-suppression, given that adherence before suppression improved for over 80% of participants. We could not perform further statistical analysis due to the small study sample size. Some patients had lower confirmatory VLs than their initial failing VL, which may be due to improved adherence. Although lower confirmatory VL levels (still above 1000 cp/mL) were recorded for half of the patients with confirmed DRMs, a drop in VL levels may not indicate that DRMs are not being developed as mutant viruses may have a fitness cost, resulting in lower viral replication capacity and/or hyper-susceptibility to other ARVs [10].

Given that this cohort re-suppressed VL after confirmation of VF and in the absence of a drug switch, it is not surprising that they had low levels of DRMs and only 23% had confirmed resistance to at least one drug. Our findings reiterate the utility of VL and DRM monitoring, as reported in other studies, and show these may be best utilised in combination with the adherence profile especially when considering drug switch [11–13]. An evaluation of adherence patterns in patients with confirmed VF may be necessary before deciding to switch/change regimen.

Patients suspected to be failing treatment clinically are required to undergo intensified adherence counselling while the result of repeat VL is awaited [14–16]. Counselling reinforces the importance of adherence, for both the 1 L and the 2 L, in case the patient is eventually switched, as poor adherence is considered to be a major driver of 2 L treatment failures [17]. Several factors influence patients’ adherence to therapy and these factors are consistent across different economic settings [18, 19]. Reported barriers to adherence include HIV-associated stigma, forgetfulness, complicated regimen, and falling asleep, whereas facilitators of adherence include simplified regimen, understanding the need for adherence, having an adherence partner and use of reminder tools [4, 18]. In this cohort with 51% married, if the couples are sero-concordant and have disclosed, they could serve as adherence partners for each other.

There are limitations in this study. Firstly, the population were selected retrospectively from electronic medical records and may not be representative of the population. Secondly, we are not certain why these patients with confirmed VF were not switched at the various ART centres and if adherence was considered at the time. Given this is a retrospective study, data were not captured on the reasons why each patient was not switched. It is possible patients were not switched due to other issues, such as logistics challenges or delays in data availability for clinical decision-making. Secondly, the small sample size, due to over half of the samples failing genotyping, reduced the power for statistical analysis outcomes. The failure to genotype might be caused by the degradation of RNAs in the stored plasma samples.

## Conclusions

Despite the limitations of the study, our results reveal that in the absence of a regimen switch, patients who re-suppressed their VL following confirmed virologic failure had few underlying DRMs and remained largely susceptible to the current 1 L regimens. We surmise that when consistent adherence is not assured, patients could exhibit virologic failure, with two VLs above 1000cp/mL, without developing mutations associated with drug resistance.

## Methods

### Study design and sites

As part of a larger evaluation on DRMs in patients experiencing virologic failure (VF) [20], we conducted a retrospective cohort study utilizing stored data and samples from patients who had been receiving ART at three large tertiary treatment centres affiliated with the Harvard/APIN Public Health Initiatives (PHI), the Centers for Disease Control and Prevention-funded United States President’s Emergency Plan for AIDS Relief (PEPFAR) Program in Nigeria: the Nigerian Institute of Medical Research (NIMR), Jos University Teaching Hospital in Jos (JUTH), and University College Hospital in Ibadan (UCH). With PEPFAR funding, all three comprehensive treatment centres have been performing VL routinely since 2004, have capacities for routine − 80 °C sample storage and for sequencing HIV in-country. An electronic medical record system (EMRS) has been utilized to record demographic, medical history, pharmacy pickups, laboratory and clinical data of each patient as well as consent for programmatic and/or for research use of samples/data [21]. At Harvard/PHI PEPFAR sites in Nigeria, clinicians in facilities with electronic medical records use the pharmacy refill data to assess adherence.

### Study population

Adult ART patients with the following characteristics were included in the study: 1) provided consent for use of data and samples in future research studies; 2) received 1 L ART, either AZT + 3TC + NVP/EFV or TDF + 3TC/FTC + NVP/EFV, between the years of 2004–2009 for at least 6 months; 3) met World Health Organisation VF criteria for 1 L treatment failure (two consecutive VL measurements greater than 1000 cp/mL); and 4) re-suppressed VL to ≤400 cp/mL following confirmation of VF. These patients may not be representative of their various population being selected from electronic medical records for meeting the criteria above. In addition, we report here findings from the second aim of a study, see Additional file 1 for more details on the study population. Findings from aims one cohort have been earlier reported [19]. The cohort in aims one were patients who failed, did not re-supress viral load on 1 L regimen and were subsequently switch to second-line regimen.

### Laboratory methods

Data on VLs were accessed from existing clinical databases (EMRS). VL were earlier determined using the Roche Cobas Amplicor Monitor assay, version 1.5 (Roche Diagnostics, Branchburg, NJ, USA) and the Roche Cobas Ampliprep/COBAS TaqMan HIV-1 Test, v2.0 kits (Indianapolis, IN, USA). Stored frozen plasma samples collected from patients who met the inclusion criteria were retrieved for HIV drug resistance testing using ATCC® HIV-1 Drug Resistance Genotyping Kit [22] (American Type Culture Collection, Manassas, VA, USA). We analysed the samples collected at the initial unsuppressed VL time point (first high VL, F_1_). If the first attempt for genotyping was not successful, a plasma sample collected at the confirmatory VF time point (F_C_) was used for repeat genotyping. In brief, HIV ribonucleic acid (RNA) was extracted from the plasma samples using the Qiagen Viral RNA Kit (Qiagen Inc., Valencia, CA, USA). Reverse transcriptase-polymerase chain reaction (RT-PCR) and nested PCR were performed using the ATCC® HIV-1 Drug Resistance Genotyping kit module 1. The nested PCR products were purified using ExoSAP-IT enzyme and used for cycle sequencing with the kit module 2. The sequencing was performed with Genetic Analyser 3130xL (Applied Biosystems, Foster City, CA, USA) and AB1 files were used to generate consensus sequences using ReCall 2.25 software (University of British Columbia, Vancouver, BC, Canada). Sequence identity matrices were performed using BioEdit software (Ibis Biosciences, Carlsbad, CA, USA) to check for contamination and the quality-confirmed sequence files were analysed with Stanford HIVDB Calibrated Population Resistance “QA details” to confirm base calls [23].

### HIV DRM interpretation and impact on drug regimens

HIV DRMs and profiles were determined using HIVdb algorithm version 8.2 [24] at the Stanford HIVDB website. To analyse the impact of DRMs on the efficacy of the potential 2 L regimens on those patients carrying drug resistant viruses, the genotype sensitivity score (GSS) was calculated per individual drug and compiled to obtain a GSS for each patient [25]. The GSS for each drug in the regimen were assigned as follows: susceptible = 1.0, potential low-level resistance = 0.75, low-level resistance = 0.5, intermediate resistance = 0.25, and high-level resistance = 0.0. HIV-1 subtyping used the REGA HIV-1 subtyping tool - version 3.0 (University of Pretoria, Pretoria, Gauteng South Africa and the REGA Institute, Katholieke Universiteit Leuven, Leuven, Belgium) on the newly obtained sequences.

### Statistical analyses

Patient characteristics at ART initiation, including estimated adherence and VL measurements were examined using univariate methods. Adherence was estimated as medicine possession ratio (MPR) using pharmacy drug refill data. MPR was computed by dividing the total number of pills provided by the number of days in the period between drug pick-ups and then multiplied by 100. Average adherence was computed for the time from ART initiation to F_1_, time from F_1_ to F_C_, and time from F_C_ to viral re-suppression. Bivariate methods were used to examine the relationship between patient characteristics and drug resistance using Epi Info software. ANOVA or Wilcoxon Two-Sample Test (Kruskal-Wallis test) was used for continuous variables while chi-squared or Fisher’s exact tests was used for categorical variables as appropriate. The deidentified study database is available as Additional file 2.

### Ethical approval

Ethical approval for this study was obtained from ethics committees of NIMR, JUTH, UCH and the Harvard T. H. Chan School of Public Health. The study was reviewed according to the Centers for Disease Control and Prevention (CDC) human research protection procedures and was approved as research, but CDC was not engaged. At these tertiary facilities, HIV-positive persons at enrolment either deny access or provide written consent for further use of their samples for research purposes. Only patients who consented and gave documented approval for use of their samples for research purposes were included in this study.

## Supplementary information

**Additional file 1.** Study Population. Gives greater details about the study population and how the participants were selected.

**Additional file 2.** Study Database. Unlinked database without personal identifiable data.

## Data Availability

The sample sequences have been submitted to GenBank with submission number BankIt1960888 and accession numbers MF684375-MF684431. Database supporting the conclusion of this study is included as additional file: Additional File 1: Study Database. Database containing deidentified clinical and laboratory data of participants.

## Abbreviations

1 L: First-line
2 L: Second-line
3TC: Lamivudine
ART: Antiretroviral therapy
ARV: Antiretroviral drugs
ATV/r: Atazanavir/ritonavir
AZT: Zidovudine
CDC: Centers for disease control and prevention
DRM: Drug resistance mutation
EFV: Efavirenz
EMRS: Electronic medical record system
F_1_: First viral load above 1000 copies/mL
F_C_: Another viral load above 1000 copies/mL confirming virologic failure
FTC: Emtricitabine
GSS: Genotype sensitivity score
IQR: Interquartile range
JUTH: Jos University Teaching Hospital, University of Jos
LPV/r: Lopinavir/ritonavir
MPR: Medicine possession ratio
NFV: Nelfinavir
NIMR: Nigerian institute of medical research
NNRTI: Non-nucleoside reverse transcriptase inhibitor
NRTI: Nucleoside reverse transcriptase inhibitor
NVP: Nevirapine
PCR: Polymerase chain reaction
PEPFAR: United States president’s emergency plan for aids relief
PI: Protease inhibitor
RPV: Rilpivirine
TDF: Tenofovir
UCH: University college hospital, university of ibadan
VF: Virologic failure
VL: Viral load
