# Supplementary material for: Low levels of HIV-1 drug resistance mutations in patients who achieved viral re-suppression without regimen switch: a retrospective study
Source: BMC Microbiol. 2020 Jan 20;20:17. doi: 10.1186/s12866-020-1706-1 (PMC6971913; doi:10.1186/s12866-020-1706-1)
Supplement: Supplementary file 1 — Additional file 1. Study Population. Gives greater details about the study population and how the participants were selected. [file 12866_2020_1706_MOESM1_ESM.pdf]

4.0 STUDY POPULATION AND SITES

4.1 Background on Study Population

The proposed study will utilize data and samples from patients that have received ART at 3 large tertiary treatment centers currently affiliated with the APIN PEPFAR Program in Nigeria and have certified laboratories with genotyping capabilities, which is unique in RLS. The three sites, namely NIMR, JUTH, and UCH, are located in geographically distinct regions across the country. JUTH is North UCH is in the South, and NIMR is located in Lagos, a cosmopolitan city with a highly heterogeneous population. Through PEPFAR funding, all 3 treatment centers have been providing comprehensive HIV care services for over 8 years, currently supplying 21,138 patients with ART (Table 2). They were chosen for this study because they are located in geographically distinct areas and provide a broad surveillance area across the country.

Table 2. ART Patients Currently Enrolled at 3 Study Sites

Indicators	Site		
	NIMR	JUTH	UCH
Number of adult patients currently on ART	7,073	8,596	5,469
Number currently receiving 2L regimen (subset of total)	842	993	838

4.2 Inclusion and Exclusion criteria

Using electronic patient data collected between the years 2004-2013, a cohort of patients from each of the three sites will be identified for inclusion, based on the following criteria: 1) previously ART-naïve adult patients who initiated either AZT+3TC+NVP/EFV or TDF+3TC/FTC+NVP/EFV for their 1L regimens in the APIN/Harvard PEPFAR program; 2) patients who meet current WHO virologic failure criteria (two consecutive viral load (VL) measurements greater than 1,000 copies/mL after at least 6 months on 1L ART); and, 3) patients who have previously consented for use of their data and samples for research.

Exclusion criteria include: 1) patients that met the criteria for virologic failure, showed adequate adherence, but were never switched to 2L or resuppressed; and, 2) patients that were switched to 2L that do not have both M6 and M12 post-switch VL data.

Of those who have met the inclusion criteria, the sites will segregate patients into the following 3 groups: 1) patients that were switched to 2L ART following the 2 consecutive VL >1,000 copies/mL, n = 600 (300 total on AZT based 1L regimen and 300 total on TDF based 1L regimen) (**Group A**); and, 2) those who, after initial failure, suppressed (VL ≤400 cp/mL) in the absence of switching due to improved adherence, n = 201 (**Group B**; Figure 2).; and 3) those who, after initial failure, were maintained on 1L due to poor adherence, n=240 (**Group C**; Figure 2). Group C patients will be segregated into 12 (n=80), 24 (n=80) and >24 months (n=80) on non-suppressed 1L regimen, with a minimum of 80 patients per duration period.

Figure 2. Stratification of patient cohort included in the study

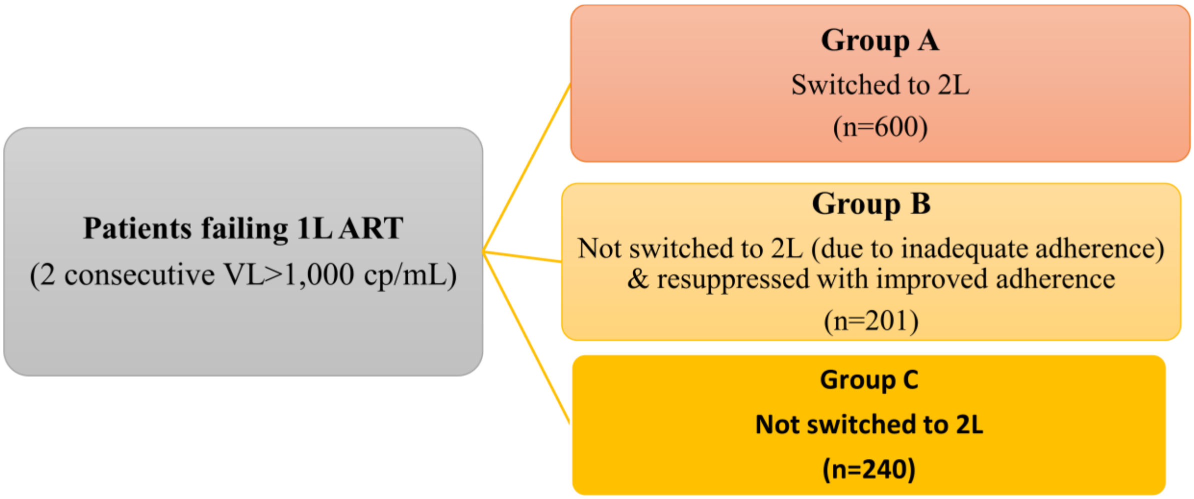

Rationale for Groups A and B.

Figure 3. Examples of virologic failure patterns seen in patients failing 1L ART in the Harvard/APIN PEPFAR Program.

a) Patient that experiences viral failure (2 consecutive VL>1,000 cp/mL) and is switched to 2L regimen due to confirmation of adequate adherence when on 1L regimen

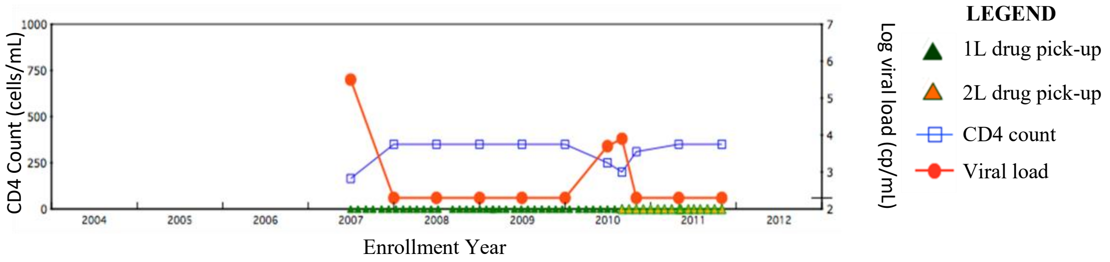

b) Patient that experiences viral failure due to inadequate adherence (note gaps in 1L drug pick-up in 2008-early 2009, green triangles) and subsequently suppresses virus with improved adherence

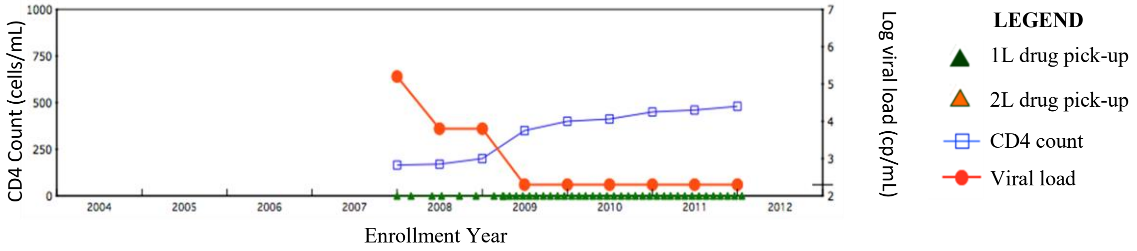

Given that we are retrospectively extracting data and samples, we expect variability in clinician decisions regarding timing for 2L switch, where some patients were switched immediately after meeting virologic failure criteria (Figure 3a), while others might have remained on a failing regimen

for some period of time until adequate adherence was demonstrated. The natural variation in ART duration will not only provide resistance data from a real-life setting, but will also allow for analysis according to duration of ART exposure and provide insight regarding the impact of accumulating multiple DRMs on subsequent 2L outcomes. Group B will help address Aim 2, as these patients and their genotypes serve as an important comparator to address the question of why some patients with sub-optimal adherence suppress after failure while others develop regimen-compromising resistance (Figure 3b).
